# Secreted Cyclic Di-GMP Induces Stalk Cell Differentiation in the Eukaryote Dictyostelium discoideum

**DOI:** 10.1128/JB.00321-15

**Published:** 2015-12-14

**Authors:** Zhi-hui Chen, Pauline Schaap

**Affiliations:** School of Life Sciences, University of Dundee, Dundee, United Kingdom

## Abstract

Cyclic di-GMP (c-di-GMP) is currently recognized as the most widely used intracellular signal molecule in prokaryotes, but roles in eukaryotes were only recently discovered. In the social amoeba Dictyostelium discoideum, c-di-GMP, produced by a prokaryote-type diguanylate cyclase, induces the differentiation of stalk cells, thereby enabling the formation of spore-bearing fruiting bodies. In this review, we summarize the currently known mechanisms that control the major life cycle transitions of Dictyostelium and focus particularly on the role of c-di-GMP in stalk formation. Stalk cell differentiation has characteristics of autophagic cell death, a process that also occurs in higher eukaryotes. We discuss the respective roles of c-di-GMP and of another signal molecule, differentiation-inducing factor 1, in autophagic cell death *in vitro* and in stalk formation *in vivo*.

## INTRODUCTION

The cyclic nucleotides cyclic AMP (cAMP) and cGMP have been known for almost 60 years to acts as intracellular second messengers across most domains of life (1). Second-messenger functions for the dimeric forms c-di-GMP, c-di-AMP, and c-GMP-AMP were more recently uncovered in the prokaryote domain (2–4). However, especially c-di-GMP is now believed to be the most commonly used signaling molecule in bacteria. c-di-GMP very broadly mediates a range of cellular responses to environmental stimuli but has a particularly prominent role in triggering the changes that cause bacteria to shift from a swarming planktonic state to a sessile biofilm-associated lifestyle (5, 6).

Roles for cyclic dinucleotides in eukaryotes emerged only recently. The social amoeba Dictyostelium discoideum was shown to use c-di-GMP as a secreted signal to induce stalk formation in its multicellular fruiting bodies (7). In mammals, both c-di-GMP and the novel molecule 2′3′-cGAMP were found to activate the innate immune system by binding to STING (stimulator of interferon genes). c-di-GMP enters mammalian cells through infection with bacteria, while 2′3′-cGAMP is endogenously produced by cGMP-AMP synthase (cGAS) in response to invasion with foreign DNA, which directly activates cGAS (8, 9).

The role of c-di-GMP in D. discoideum has raised many questions about its mode of action and its interaction with other signal molecules that control the developmental program. In this review, we summarize the processes leading to fruiting body formation and discuss the specific role of c-di-GMP in stalk cell differentiation.

## THE DICTYOSTELIUM LIFE CYCLE IS DOMINATED BY cAMP SIGNALING

The dictyostelid social amoebas are members of the eukaryote kingdom *Amoebozoa*, which contains mostly unicellular amoebas or amoeboflagellates (10). Unlike their unicellular relatives, the *Dictyostelia* display a form of colonial multicellularity in which cells aggregate to form a multicellular fruiting structure. This life cycle is by no means unique; it is also displayed by other protists, such as Acrasis rosea in Excavata (11) and Fonticula alba (12) in Opisthokonta, and by the myxobacteria in the prokaryote domains (13).

**FIG 1:**
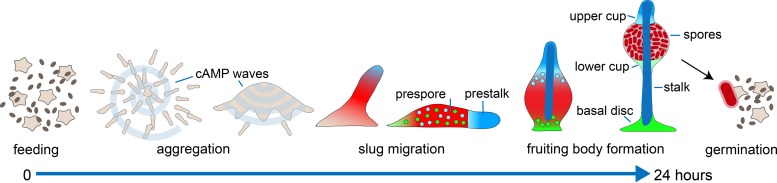
The life cycle of D. discoideum. In the course of their 24-h life cycle, starving D. discoideum amoebas aggregate by secreting pulses of the chemoattractant cAMP. Cells differentiate into spores and a number of somatic cell types that will form a stalk to lift the spores, upper and lower cups to support the spore mass, and a basal disc to support the stalk. After dispersal, the spores germinate in the presence of bacteria and resume feeding. See the text for details.

D. discoideum became the model dictyostelid after the discovery that it uses cAMP as a chemoattractant for aggregation (14). In addition to this role, cAMP appeared to have many functions both as a secreted signal and as a second messenger in controlling the Dictyostelium developmental program. Dictyostelium amoebas feed on bacteria in forest leaf litter. They initiate their multicellular life cycle in response to starvation and the accumulation of quorum-sensing factors, which cause release of translational repression of cAMP-dependent protein kinase A (PKA) (15, 16). PKA activates the expression of genes that are required for aggregation, such as cell surface cAMP receptors (cARs), adenylate cyclase A (ACA), and the extracellular cAMP phosphodiesterase PdsA (17). Some starving cells start to secrete pulses of cAMP, which trigger both chemotaxis and cAMP secretion in the surrounding cells. This causes the cAMP pulses to travel as waves through the population and the cells to move together in aggregates (Fig. 1). The aggregate tip continues to emit cAMP pulses and is pushed upward by the inflowing movement of cells underneath, thus forming the sorogen or slug (18). The slug falls over and starts migrating toward light, which in nature will lead it to the soil surface. Here it projects upward to form a fruiting body consisting of a ball of spores supported by a column of stalk cells. Additional cell types differentiate to form a basal disc to support the stalk and an upper and a lower cup to support the spore mass.

Spore differentiation starts shortly after aggregation and is induced both by secreted cAMP acting on cARs and by intracellular cAMP acting on PKA (19–21). In the posterior region of the slug, a second adenylate cyclase, ACG, is translationally upregulated, which synthesizes cAMP for both cAR and PKA activation (22). The prespore cells, in turn, synthesize the chlorinated polyketide differentiation-inducing factor 1 [1-(3,5-dichloro-2,6-dihydroxy-4-methoxyphenyl)hexan-1-one] (23), here DIF, which induces the differentiation of a population of cells that express genes associated with stalk cell differentiation (24). These cells are first intermixed with prespore cells but sort out either to the front to form the stalk and upper cup or to the rear to form the lower cup and basal disc of the fruiting body (Fig. 1).

Upon initiation of fruiting body formation, the anterior cells first synthesize a central cellulose tube, enter the tube from the top, and then differentiate into vacuolated stalk cells surrounded by a cellulose wall. The prespore cells move up the stalk and mature into spores by constructing a multilayered cellulose-rich spore wall.

## SENSOR HISTIDINE KINASES CRITICALLY REGULATE SPORE AND STALK ENCAPSULATION

Slug and fruiting body formation depends on the coordinated movement of individual amoebas. However, because amoebas will become immobilized by cell walls during spore and stalk cell maturation, intensive signaling is required to make sure that maturation occurs at the right time and place. Most signals converge to regulate PKA activity, which is required for both spore and stalk cell maturation (21, 25). In fruiting bodies, cAMP for PKA activation is synthesized by ACG and a third adenylate cyclase, AcrA (26), However, cAMP levels are actually most critically regulated by the cAMP phosphodiesterase RegA (27, 28). The phosphodiesterase activity or RegA requires phosphorylation of its N-terminal response regulator domain. A range of sensor histidine kinases/phosphatases, which are the targets of signals that regulate terminal differentiation, control the phosphorylation status of RegA. Among these signals are ammonia, the product of protein degradation in starving amoebas (29), a peptide, SDF-2 (30), and an adenine analog, discadenine (31). See reference 32 for a comprehensive review.

PKA, AcrA, RegA, and a large repertoire of sensor histidine kinases/phosphatases are not only conserved in all *Dictyostelia* (33, 34) but also in unicellular amoebozoa (35) and in unrelated amoeboflagellates in the kingdom Excavata (36). These unicellular organisms form walled cysts in response to environmental stress. Recent studies show that encystation is directly triggered by cAMP acting on PKA and that RegA negatively regulates encystation (37–40). This strongly suggests that cysts are ancestral to the walled spores and stalk cells of *Dictyostelia* and that the cAMP-mediated mechanisms that control spore and stalk differentiation are evolutionarily derived from the regulation of encystation in response to environmental stimuli.

## REGULATION OF SOMATIC CELL DIFFERENTIATION BY DIF AND c-DI-GMP

Among organisms with colonial multicellularity, the *Dictyostelia* are unique in having evolved somatic cell types that are fated to die after the spores have matured. The stalk cells are the most deeply conserved somatic cells of *Dictyostelia* (41), but D. discoideum has three additional somatic cell types that make up the basal disc and the upper and lower cups of the fruiting body. DIF was originally identified as a secreted factor that triggers stalk cell differentiation in submerged monolayers of D. discoideum cells *in vitro* (42). Two DIF-inducible genes, *ecmA* and *ecmB*, were identified that encode extracellular matrix proteins (24). Both genes are expressed in the stalk, basal disc, and upper and lower cups of the fruiting body. In slugs, both genes are expressed in cells that are intermixed with prespore cells at the posterior. These cells are the precursors of the upper and lower cups and basal disc cells. *ecmA* is additionally expressed throughout the anterior prestalk region, and *ecmB* is expressed in the central core of the tip. Separate regions of the *ecmA* and *ecmB* promoters regulate these complex patterns of expression. Transcription factors such as DimB, DimA, GtaC, and MybE were identified that mediate the DIF inducibility of *ecmA* or *ecmB* expression (43–47). However, deletion of these transcription factors does not prevent stalk cell differentiation, although *dimB*^−^ and *gtaC*^−^ mutants cannot form the basal disc (47, 48). Deletion of genes encoding enzymes in the DIF biosynthetic pathway, such as the polyketide synthase StlB, the methyltransferase DmtA, and the chlorinase ChlA, also do not prevent stalk cell differentiation (49–51). However, *stlB*^−^ and *dmtA*^−^ mutants also do not form the basal disc and both slugs and stalks are structurally weaker (51).

Another stalk inducer factor was discovered by serendipity. Annotation of Dictyostelium genomes revealed the presence of single genes with a highly conserved diguanylate cyclase domain (7). This was the first putative diguanylate cyclase to be detected in eukaryotes. A search for its biological role was therefore initiated by deleting the single diguanylate cyclase gene, *dgcA*, of D. discoideum. *dgcA*^−^ mutant cells showed normal growth and development up to the slug stage but could not form fruiting bodies. Fruiting body formation was restored by mixing in 10% wild-type cells, indicating that the *dgcA*^−^ mutant lacked a signal secreted by wild-type cells. Since the prokaryote diguanylate cyclase synthesizes c-di-GMP, the missing signal was likely to be c-di-GMP. This was confirmed by the observation that fruiting body formation was restored by temporarily submerging slugs in buffer containing c-di-GMP, while c-di-AMP, GTP, GMP, and cGMP were without effect.

Fruiting body formation is a complex process that depends on the concerted movement of cells to project the cell mass upward, the synthesis of a cellulose tube, and the differentiation of the vacuolated walled stalk cells. Cellulose synthesis appeared not to be perturbed, since *dgcA*^−^ mutant slugs still synthesized cellulose in the slime sheath that surrounds the slugs. However, the *dgcA*^−^ mutant did not express genes that are specific to fully formed stalk and spore cells. Further studies showed that c-di-GMP induced the expression of the *ecmB* gene from its stalk-specific promoter region but not the expression of spore genes. The spores differentiate later than the stalk cells, and the defective spore gene expression of the *dgcA*^−^ mutant is therefore likely a derived effect. Similar to DIF, c-di-GMP also induced stalk cell differentiation directly in submerged cell monolayers. *dgcA* is itself expressed in the anterior prestalk region of the slug, which, combined with the stalk-inducing activity of c-di-GMP, strongly suggested that c-di-GMP is the signal that induces the differentiation of the stalk.

## c-DI-GMP, DIF, AND AUTOPHAGIC CELL DEATH

Stalk cell differentiation ends in cell death and is accompanied by extensive autophagy that ultimately leads to almost complete degradation of the contents of the cell (52). Stalk cell differentiation does not display the hallmarks of either apoptosis or necrotic cell death and depends on the presence of the autophagy gene *atg1* (53). It was therefore attributed to autophagic cell death (ACD) (54), a form of programmed cell death that also occurs in higher eukaryotes and has implications for cancer therapy (55).

DIF-induced ACD in Dictyostelium is, for this reason, also studied as a cell biological process because the genetic tractability of Dictyostelium offers opportunities to identify crucial components of the ACD pathway. Forward genetic studies have identified a number of proteins that are required for DIF-induced ACD in cell monolayers. One of these proteins is the inositol 3-phosphate receptor IplA (56), which mediates Ca^2+^ flux from the endoplasmic reticulum to the cytosol (57). This finding complements earlier work showing that DIF increases Ca^2+^ levels and that its effects on *ecmB* expression can be mimicked by agents, such as thapsigargin and 2,5-di-*t*-butyl-1,4-benzohydroquinone, that cause Ca^2+^ release from internal stores (58). The target of Ca^2+^ could be the protein phosphatase calcineurin, which is regulated by Ca^2+^/calmodulin, because the calcineurin inhibitor cyclosporine prevents DIF-induced ACD (56). Other essential proteins for DIF-induced ACD are talin B (54), a cytoskeletal protein that links the actin cytoskeleton to cell adhesion (59) and the sensor histidine kinase DhkM (60). As described above, Dictyostelium histidine kinases control intracellular cAMP levels and PKA activation mainly by regulating the activity of the cAMP phosphodiesterase RegA. ACD could be restored in *dhkM*^−^ mutant cells by the PKA activator 8Br-cAMP, suggesting that DhkM acts as a phosphatase to inhibit RegA activity (60).

Surprisingly, neither IplA, DhkM, nor Talin B was required for induction of ACD by c-di-GMP, and c-di-GMP induced ACD was also not sensitive to cyclosporine (61). Additionally, while DIF induces nuclear translocation of the transcription factor DimB, this was not the case for c-di-GMP. It was concluded that c-di-GMP and DIF use different signal transduction pathways to induce ACD (61). However, the results obtained with DimB actually indicate that the phenotypically similar forms of ACD that are induced by c-di-GMP or DIF represent two distinct differentiation pathways. In normal development, DimB is enriched in the nuclei of a subpopulation of cells in the prespore region that will give rise to the basal disc and lower cup of the fruiting body. Deletion of DimB prevents the differentiation of these cell types, but *dimB*^−^ mutant cells still form normal stalks (48). As described above, loss of StlB and DmtA, two enzymes required for DIF synthesis, leaves stalk formation intact but prevents differentiation of the basal disc and lower cup (51). Because basal disc and stalk cells have the same vacuolated walled phenotype, this means that DIF-induced ACD in monolayers actually represents basal disc differentiation, while c-di-GMP-induced ACD represents stalk cell differentiation. There are, as yet, no markers for basal disc-specific gene expression, and the two processes therefore cannot be distinguished by *in vitro* experiments.

When added together, DIF and c-di-GMP are more effective at inducing ACD than each stimulus is on its own. Induction is more rapid, and a larger number of vacuolated cells is being induced, suggesting a synergistic effect of c-di-GMP and DIF. DIF appears to be required for c-di-GMP-induced ACD, since c-di-GMP-induced ACD is absent from or strongly reduced in *stlB*^−^ and *dmtA*^−^ mutants. Conversely, DIF still induces ACD in the *dgcA*^−^ mutant, indicating that the DIF response does not require c-di-GMP (61).

We recently identified several c-di-GMP-inducible stalk genes from transcriptomic profiling of *dgcA*^−^ mutant and wild-type multicellular structures. In monolayers of wild-type cells, these genes are optimally induced by 1 μM c-di-GMP and not by DIF. In either the *dmtA*^−^ or the *stlB*^−^ mutant, at least 10-fold higher c-di-GMP concentrations are required for stalk gene expression. This suggests that DIF promotes responsiveness to c-di-GMP (Chen and Schaap, unpublished data), thus explaining the synergistic effect of DIF and c-di-GMP on ACD. Remarkably, the absence of DIF does not impede stalk cell differentiation in normal development (51), while c-di-GMP is essential (7). This suggest that, within multicellular structures, other signals may induce responsiveness to c-di-GMP. DIF evidently replaces these signals when ACD is induced in monolayers.

**FIG 2:**
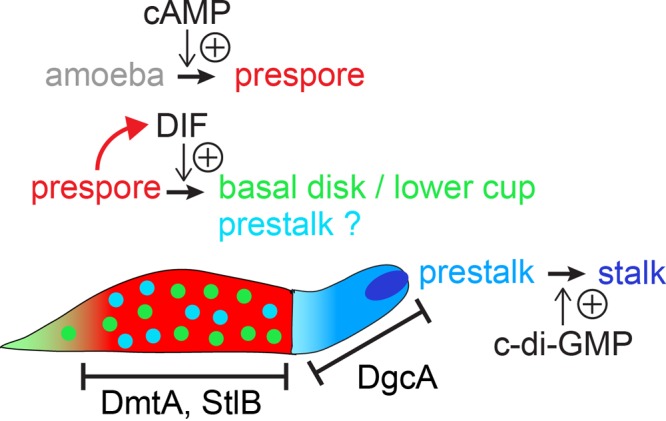
Model of the roles of DIF and c-di-GMP in Dictyostelium development. cAMP produced by ACG in the slug posterior induces the differentiation of prespore cells (22). The prespore cells, in turn, express DmtA and StlB, two enzymes in the DIF-synthetic pathway (49, 51). DIF causes the dedifferentiation of prespore cells into (pre)basal disc and lower cup cells (51). DgcA is expressed in the anterior prestalk region of the slug and induces the transition from prestalk to stalk differentiation (7). It is unclear to what extent DIF is responsible for differentiation of the anterior prestalk cells. *dmtA*^−^ mutants but not *stlB*^−^ mutants show a reduced anterior prestalk region (49, 51). The difference between *dmtA* and *stlB* (which is more upstream in DIF synthesis) was considered to be due to the accumulation of des-methyl-DIF in the *dmtA*^−^ mutant, since des-methyl-DIF inhibits prestalk differentiation (51).

The observed effects of c-di-GMP and DIF on cell type specification and the localization of the enzymes that synthesize both compounds indicate specific roles for each compound (Fig. 2). DIF, synthesized by prespore cells in the rear of the slug, induces differentiation of prebasal disc and lower cup cells, while c-di-GMP produced by prestalk cells in the front causes the transition of prestalk cells to stalk cells. The question remains to what extent DIF is responsible for the differentiation of anterior prestalk cells.

## FUTURE DIRECTIONS

There are many open questions in understanding the role of c-di-GMP in Dictyostelium development and its interaction with other signal molecules. Most importantly, there is no current information on the receptors that detect c-di-GMP and the subsequent processes that lead to the expression of stalk-specific genes. Unlike prokaryotes, where c-di-GMP acts intracellularly, Dictyostelium uses c-di-GMP as a secreted signal. Because of its negative charge and relatively large size, c-di-GMP is unlikely to be membrane permeant and probably requires integral plasma membrane receptors for detection. This renders the pull-down method, based on a c-di-GMP-specific capture compound, which was successfully used to identify bacterial c-di-GMP binding proteins (62, 63), more challenging. Forward genetics by tagged mutagenesis is a powerful tool for identifying unknown genes in developmental processes in Dictyostelium (64), and we are currently using this approach to select mutants with a cell-autonomous stalk-defective phenotype that are likely to be defective in genes involved in c-di-GMP signal processing.

Of further importance is the regulation of diguanylate cyclase itself. The *dgcA* gene is expressed throughout the prestalk region, but stalk cell differentiation initiates only in the core of the tip. This firstly raises the question of the signals that control *dgcA* expression and secondly suggests the possibility that DgcA activity is itself under regulation. Alternatively, c-di-GMP could be interacting with other signals, such as ammonia, to cause the strict position dependency of stalk formation.
